# Prognostic value of circulating Chromogranin A in prostate cancer: a systematic review and meta-analysis

**DOI:** 10.3389/fonc.2025.1521558

**Published:** 2025-02-05

**Authors:** Xiaoying Tang, Zhenyu Liu, Liangdong Song, Huixuan Zhu, Shuai Su, Delin Wang

**Affiliations:** Department of Urology, the First Affiliated Hospital of Chongqing Medical University, Chongqing, China

**Keywords:** prostate cancer, circulating Chromogranin A, neuroendocrine, meta-analysis, prognostic

## Abstract

**Background:**

There are discrepancies between the results of different studies regarding the prognostic role of circulating Chromogranin A (CgA) in prostate cancer. Therefore, we conducted a meta-analysis of the available findings to explore the value of circulating Chromogranin A in the prognosis of prostate cancer.

**Methods:**

We systematically searched the PubMed, Embase, Web of Science, Cochrane Library, and Clinical Trials databases for studies on the relationship between CgA and survival outcomes in prostate cancer from inception until December 2024, and we focused on articles detecting circulating CgA, with the primary endpoints of the studies being overall survival (OS), and progression-free survival (PFS).

**Results:**

Of the 2049 articles retrieved, 10 articles met our inclusion criteria, involving a total of 1445 patients. Elevated circulating CgA was associated with poorer OS (HR=1.82, 95% CI: 1.38–2.41; p<0.001) and PFS (HR=2.04, 95% CI: 1.42–2.94; p<0.001). However, no correlation was found between post-treatment circulating CgA changes and OS (HR=0.95, 95% CI: 0.66–1.37; p=0.767).

**Conclusion:**

Circulating CgA is a predictive marker of poor survival outcomes in prostate cancer However, the sample size of the current study is small and larger studies are needed to further validate this in the future.

## Introduction

In the United States, prostate cancer is the most diagnosed cancer (excluding non-melanoma skin cancers) and is the second leading cause of cancer deaths among men in the U.S. The cancer incidence of prostate cancer is increasing by 3% per year from 2014 through 2019. It is estimated that more than 280,000 men were diagnosed with prostate cancer in 2023 and more than 34,000 died from prostate cancer (1, 2).

Adenocarcinoma accounts for 90–95% of the pathological staging of prostate cancer and is characterized by androgen receptor (AR) and prostate-specific antigen (PSA) expression (3, 4). In the development of prostate cancer, cell survival is dependent on androgens and androgen receptors, therefore, androgen deprivation therapy (ADT) combined with anti-androgen therapy is widely used in prostate cancer treatment (5). Prostate cancer patients initially respond to hormone therapy, but the duration of the response lasts anywhere from a few months to a few years, eventually leading to castration-resistant prostate cancer (CRPC) (6).

The predominant subtype in CRPC is AR-positive adenocarcinoma (CRPC-adeno), but approximately 10–17% of patients treated with ADT or anti-androgen therapy exhibit a neuroendocrine differentiation (NE) phenotype, and these tumors are classified as neuroendocrine prostate cancer (NEPC) (4, 7). New-onset neuroendocrine prostate cancer (NEPC) is rare, accounting for under 2% of diagnosed prostate cancers. Neuroendocrine prostate cancer is characterized by the expression of neuroendocrine markers, such as Chromogranin A (CgA), synaptophysin (SYP), and neural cell adhesion molecules (CD56). It also exhibits deficiency in androgen receptor (AR) and prostate-specific antigen (PSA), and NEPC tends to be more aggressive, has a poorer prognosis, and is associated with hormone therapy resistance (4, 7).

There are different views on the origin of prostate cancer NE cells, with one view being that they are derived from pluripotent stem cells. Benign prostate tissue includes epithelial cells, including secretory epithelial cells, basal cells, and neuroendocrine cells, the first two of which are the major components of the prostate epithelium, with neuroendocrine cells accounting for approximately 1% of the entire epithelial cell population. Although these three types of cells markedly differ in marker expression and hormone regulation, they share a common origin as pluripotent stem cells, and it is a current view that prostate cancer NE cells are derived from pluripotent stem cells (4, 8). Another view is that prostate cancer NE cells arise from prostate cancer lineage plasticity. Prostate adenocarcinoma cells lose their adenocarcinoma characteristics and develop a neuroendocrine phenotype during androgen deprivation therapy (ADT), suggesting that it is driven by lineage plasticity (4, 9, 10). Although the source of NE cells in prostate cancer is difficult to determine and more studies are needed to further clarify it in the future, neuroendocrine differentiation (NED) correlates with tumor progression, and the measurement of serum NE markers can objectively respond to the neuroendocrine differentiation of tumor cell populations, among which CgA is a widely used serum marker.

Chromogranin A (CgA) is an acidic, hydrophilic secretory protein with a molecular weight of 48 kD (11) that belongs to the granin family (12). It was first found in the secretory granules of adrenal chromaffin granulocytes, which are widely distributed in the neuroendocrine system, including normal tissues and tumor tissues, and involved in energy metabolism, immunoregulation, tissue repair, and other processes. It is suggested that circulating CgA is associated with positive immunohistochemical CgA expression (13) and may serve as a supplement to PSA and provide important information on prostate cancer disease prognosis (14, 15). In contrast, other studies conclude that there is no correlation between circulating CgA and prostate cancer prognosis (16, 17).

Controversy exists regarding the value of circulating CgA in prostate cancer prognosis. Therefore, this study aimed to meta-analyze published results to investigate whether circulating CgA can provide useful prognostic information in prostate cancer.

## Methods

### Search strategy

This systematic review and meta-analysis is reported in accordance with the Preferred Reporting Items for Systematic Reviews and Meta-Analysis (PRISMA) statement (18). And we are registered in the International prospective register of systematic reviews(ID: CRD42023492830). A systematic search of the PubMed, Embase, Web of Science, Cochrane Library, and Clinical Trials databases for articles on the prognostic role of Chromogranin A (CgA) in prostate cancer was performed from inception through December 2024. We searched the following combination of Medical Subject Headings (Mesh) and related keywords: “Prostatic Neoplasms [Mesh] or Prostate Neoplasms or Prostate Cancer or Prostatic Cancer” andC “hromogranin A or CgA or CHGA”.

### Inclusion and exclusion criteria

We developed inclusion criteria based on the PICOS principles (P: population, I: intervention, C: comparison, O: outcome, S: study design): (1) Population: Patients diagnosed with prostate cancer by histopathologic examination; (2) Intervention: To detect CgA during the course of a disease, we focused on studies that have detected serum or plasma CgA; (3) Comparison: Comparing patients with elevated CgA to patients without elevated CgA to study the impact on survival outcomes; (4) Outcome: The study endpoints were overall survival (OS), progression-free survival (PFS), and the results were presented as hazard ratio (HR) and corresponding 95% confidence interval (CI); (5) Study design: We have no restrictions on the article study design. The exclusion criteria were as follows: no outcome metrics, no study of the correlation between CgA and survival outcomes in prostate cancer, as well as reviews, conference abstracts, commentaries, letters, and animal testing were excluded.

### Quality assessment and data extraction

Titles and abstracts were reviewed by two independent researchers, articles that met the inclusion criteria were retrieved in full text, and quality assessment and data extraction were completed. The decision was made after discussion with a third researcher if there was a disagreement between them.

Two independent researchers extracted the following data from the article based on a pre-designed table: Authors, publication date, country, study design, number of patients, age, CgA values, and disease stage,outcome indicators.The quality of included studies were assessed using the Newcastle-Ottawa Scale (NOS) (a score of 7–9 is considered a high-quality study, a score of 4–6 is a medium-quality study, and a score of less than 4 is considered a low-quality study). Differences between the two researchers were resolved by consensus.

### Statistical analysis

We performed a meta-analysis using Stata (version 15.0) to pool the results of the included studies to calculate the overall hazard ratio (HR) and their 95% confidence interval (CI). Assessing heterogeneity across studies using the I^2^ test (cochrane classification: 0% to 40%: might not be important, 30% to 60%: may represent moderate heterogeneity, 50% to 90%: may represent substantial heterogeneity, 75% to 100%: considerable heterogeneity). A fixed-effects model was used if I^2^ <50%, while a random-effects model was used if I^2^ >50%. Meta-regression and subgroup analysis were performed to find sources of heterogeneity when it was apparent. We evaluated publication bias using the Egger’s test, which suggests the presence of publication bias if the P value is <0.05. Sensitivity analysis were performed using the trim-and-fill method to assess the robustness of the results. Of the 10 papers included, Patients in the Szarvas et al. study (19) were divided into three cohorts based on treatment modalities, comprising radical prostatectomy cohort, docetaxel cohort, and abiraterone/enzalutamide cohorts. The radical prostatectomy cohort was excluded from our study due to different study outcomes. The other two cohorts existed independently of each other as separate observation cohorts. Therefore, the docetaxel cohort and abiraterone/enzalutamide cohorts were considered as two separate outcomes to be combined in the summary analysis of results.

## Result

**Figure 1:**
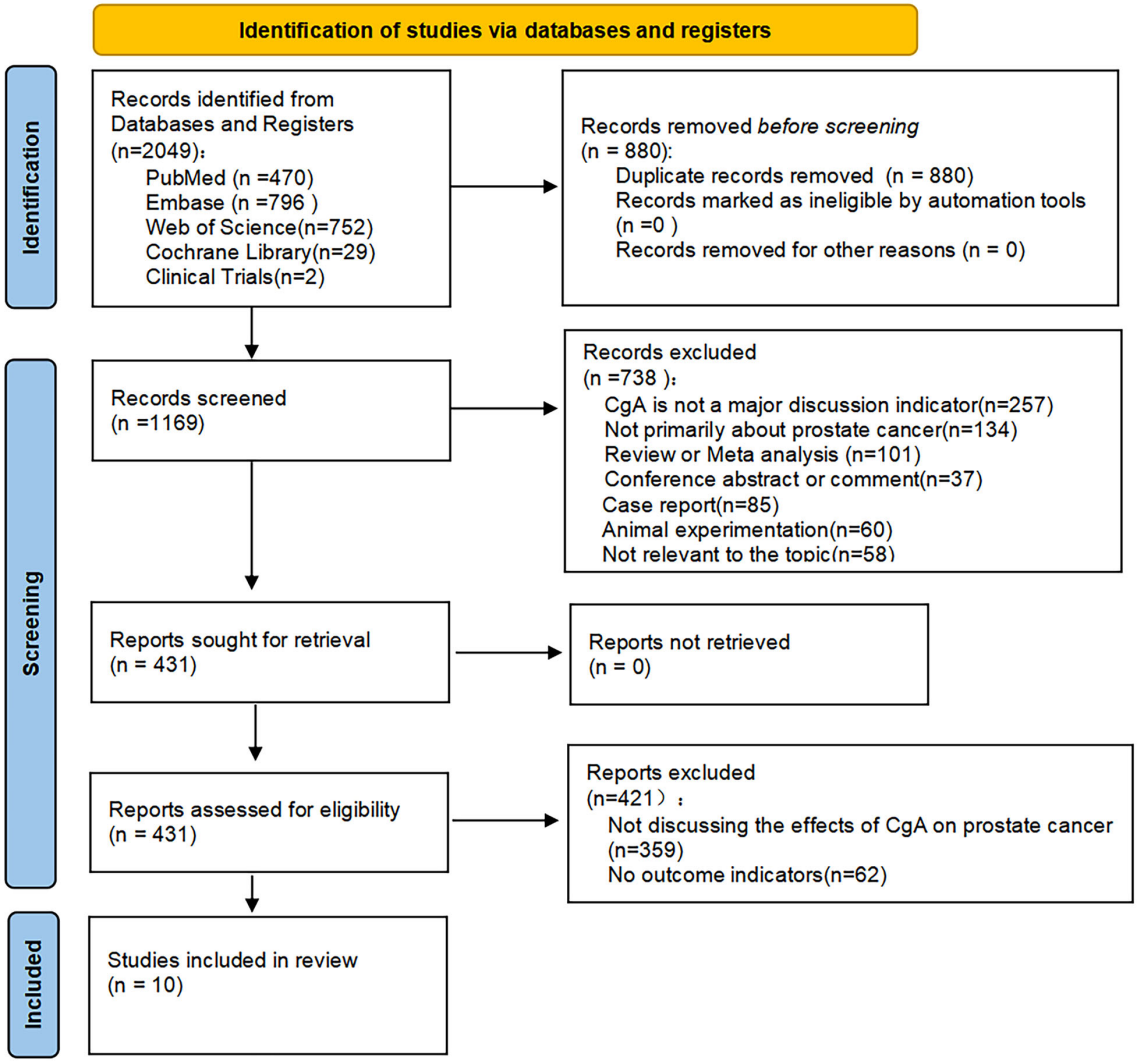
PRISMA flow diagram of study identification and inclusion process.

By searching several databases, a total of 2049 studies were retrieved, 880 duplicates were excluded, and a total of 738 studies were excluded by reading the titles and abstracts for the following reasons: Not primarily about CgA or prostate cancer, not consistent with research topic, reviews, meeting abstracts, comments, letters, and animal testing, 421 of the remaining studies were excluded because they did not discuss the relationship between CgA and prostate cancer prognosis or had no relevant outcome indicators. A total of 10 articles were eventually included in our study (17, 19–27) (**Figure 1**).

### Study characteristics and quality assessment

A total of 10 studies involving 1,445 patients from 4 countries were included (17, 19–27). Articles were published between 2004 and 2023, with five studies from Italy, three from the United States, one from Hungary, and one from Germany. A total of 7 retrospective studies and 3 prospective studies were used with the median age of patients between 70–75 years old. Ten study outcomes were overall survival (OS) and four were progression-free survival (PFS), with two studies looking at the impact of post-treatment CgA changes on survival outcomes. We evaluated the quality of the included studies using the Newcastle-Ottawa Scale (NOS), and all studies scored between 7 and 9 as high-quality studies, **Table 1** shows the characteristics of each included study (**Table 1**).

**Table 1:** Characteristics and quality scores of included studies.

Year	Country	Study design	Sample size	Median age (year)	Median CgA	Disease stage	Treatment	Outcome	Quality scores
2007	Italy	Retrospective cohort	175	70	11.3U/L	PC	LHRHa	OS	9
2018	USA	Retrospective cohort	200	72	100.3ng/ml	mCRPC	ADT	OS	8
2018	Italy	Retrospective cohort	197	73	122.0ng/ml	CRPC	ADT	PFS,OS	8
2021	Hungary	Retrospective cohort	95	71	111.1ng/ml	mCRPC	DOC	OS	7
2021	Hungary	Retrospective cohort	143	73	149.9ng/ml	mCRPC	Abi/Enza	OS	7
2005	Italy	Retrospective cohort	108	74	17.3U/L	CRPC	LHRHa	OS	8
2014	Italy	Retrospective cohort	48	73	235.5ng/ml	mCRPC	ADT	PFS,OS	8
2014	Italy	Retrospective cohort	35	75	174.0ng/ml	mCRPC	Enza	PFS,OS	8
2023	USA	Prospective cohort	71	72	83.0ng/ml	mCRPC	ADT	OS	8
2017	Germany	Prospective cohort	52	71.3	132.0ng/ml	mCRPC	DOC	PFS,OS	7
2004	USA	Prospective cohort	321	70	12.0U/L	mCRPC	Suramin	OS	8

PC, prostate cancer; CRPC, castration-resistant prostate cancer; mCRPC, metastatic castration-resistant prostate cance; LHRHa, Leuteinizing Hormone-Releasing Hormone Agonist; ADT, Androgen deprivation therapy; OS, overall survival; PFS, progression-free survival; Abi, Abiraterone; Enza, Enzalutamide; DOC, Docetaxel.

### Synthesis of results

**Figure 2:**
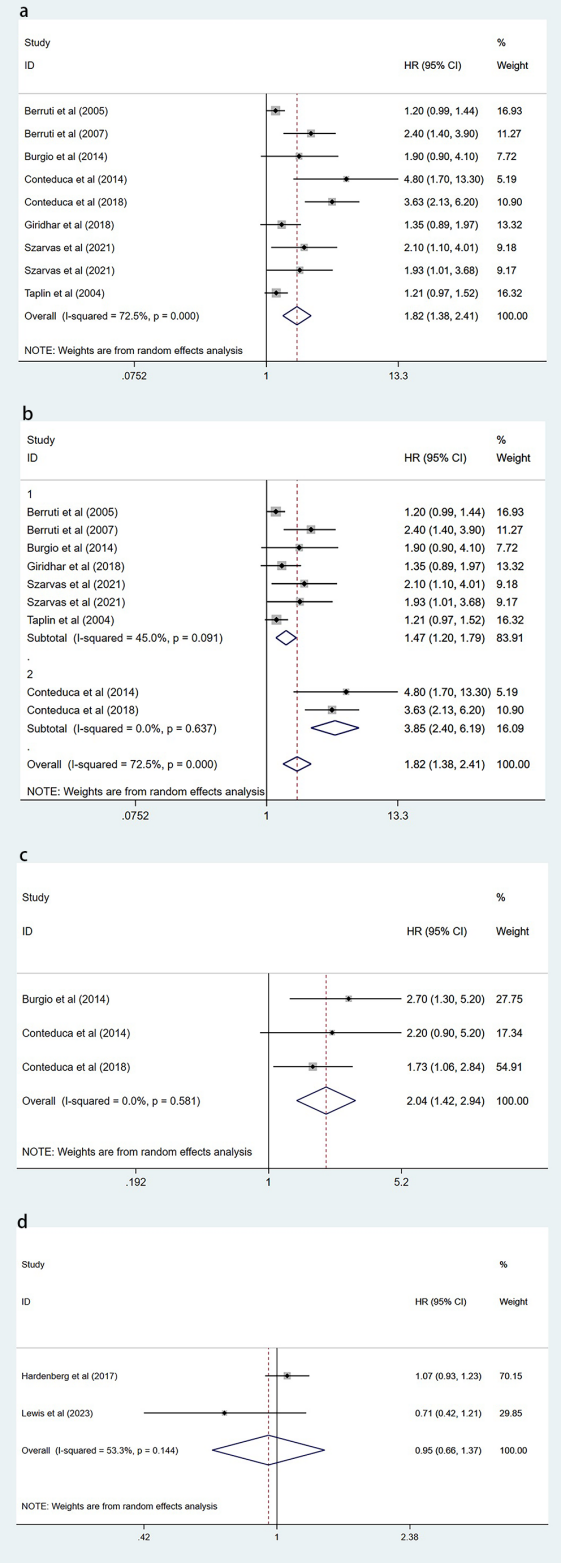
Forest plot of the effect of circulating CgA on survival outcomes. **(A)** Forest plot comparing elevated circulating CgA with OS. **(B)** Forest plot comparing elevated circulating CgA with OS in different subgroups. **(C)** Forest plot comparing elevated circulating CgA with PFS. **(D)** Forest plot comparing circulating CgA changes with OS. CgA, chromogranin A; OS, overall survival; PFS, progression-free survival; HR, hazard ratio; CI, confidence interval.

**Figure 3:**
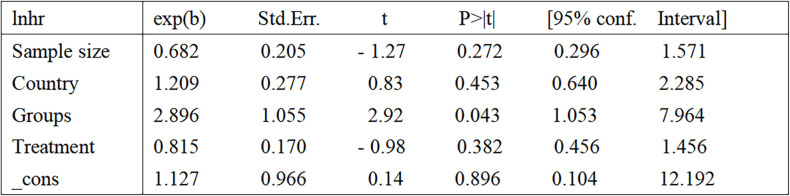
Results of meta-regression. samplesize (1=Sample size less than 100, 2=Sample size greater than 100); country (1=Italy, 2=USA, 3=Hungary); groups [1=several studies other than Conteduca et al. (17, 19–22, 25–27), 2=two studies by Conteduca et al. (23, 24)]; treatment (1=LHRHa, 2=ADT, 3=Other treatments). LHRHa, Leuteinizing Hormone-Releasing Hormone Agonist; ADT, Androgen deprivation therapy.

Eight of these papers discuss the relationship between circulating CgA and OS (17, 19–24, 26). A combination of the data suggests that elevated circulating CgA negatively correlates with OS (HR=1.82, 95% CI: 1.38–2.41; p<0.001). There was heterogeneity between studies (I^2^ = 72.5%, p <0.001); therefore, random effects models were used to combine the analysis (**Figure 2A**). We performed meta-regression and subgroup analysis to find the source of heterogeneity. In our included literature, two studies by Conteduca et al. (23, 24) compared patients with circulating CgA elevations of more than three times the upper normal limit or elevations of less than three times the upper limit of normal levels to explore the impact on survival outcomes. This grouping may have yielded more significant results compared with groupings in other studies, which may have contributed to the source of heterogeneity. Therefore, we grouped these two studies into one category and performed a multifactorial meta-regression of the sample size, different CgA grouping methods, country, and treatment modality of each study. The results suggested that the grouping method of CgA may be a source of heterogeneity (P=0.043) (**Figure 3**). We subsequently performed subgroup analysis according to the different groupings of CgA with several studies other than Conteduca et al. as subgroup 1 (17, 19–22, 25–27) and two studies by Conteduca et al. as subgroup 2 (23, 24). The results showed no significant heterogeneity between subgroup 1 studies (I^2^ = 45.0%, p=0.091) and no heterogeneity between subgroup 2 studies (I^2^ = 0.0%, p=0.637). Results in both subgroups suggest that elevated CgA is negatively associated with OS (subgroup 1, HR=1.47, 95% CI: 1.20–1.79; p<0.001; subgroup 2, HR=3.85, 95% CI: 2.4–6.19; p<0.001) (**Figure 2B**). Therefore, different ways of grouping CgA were considered possible sources of heterogeneity.

There are three publications examining the effect of elevated circulating CgA on PFS (22–24). One publication examines the impact of post-treatment CgA changes on PFS (27). Data from three articles examining the relationship between elevated circulating CgA and survival outcomes were pooled and showed that elevated circulating CgA was negatively associated with PFS (HR=2.04, 95% CI: 1.42–2.94; p<0.001) (**Figure 2C**).

Two articles examined the association between post-treatment changes in circulating CgA and OS (25, 27). The pooled results showed no correlation between post-treatment CgA elevation and OS (HR=0.95, 95% CI: 0.66-1.37; P=0.767) (**Figure 2D**). This suggested that elevated CgA after treatment does not lead to poorer OS.

The latter two outcomes were limited in subgroup analysis, sensitivity analysis, and risk of bias evaluation because of the small number of included studies.

### Publication bias

**Figure 4:**
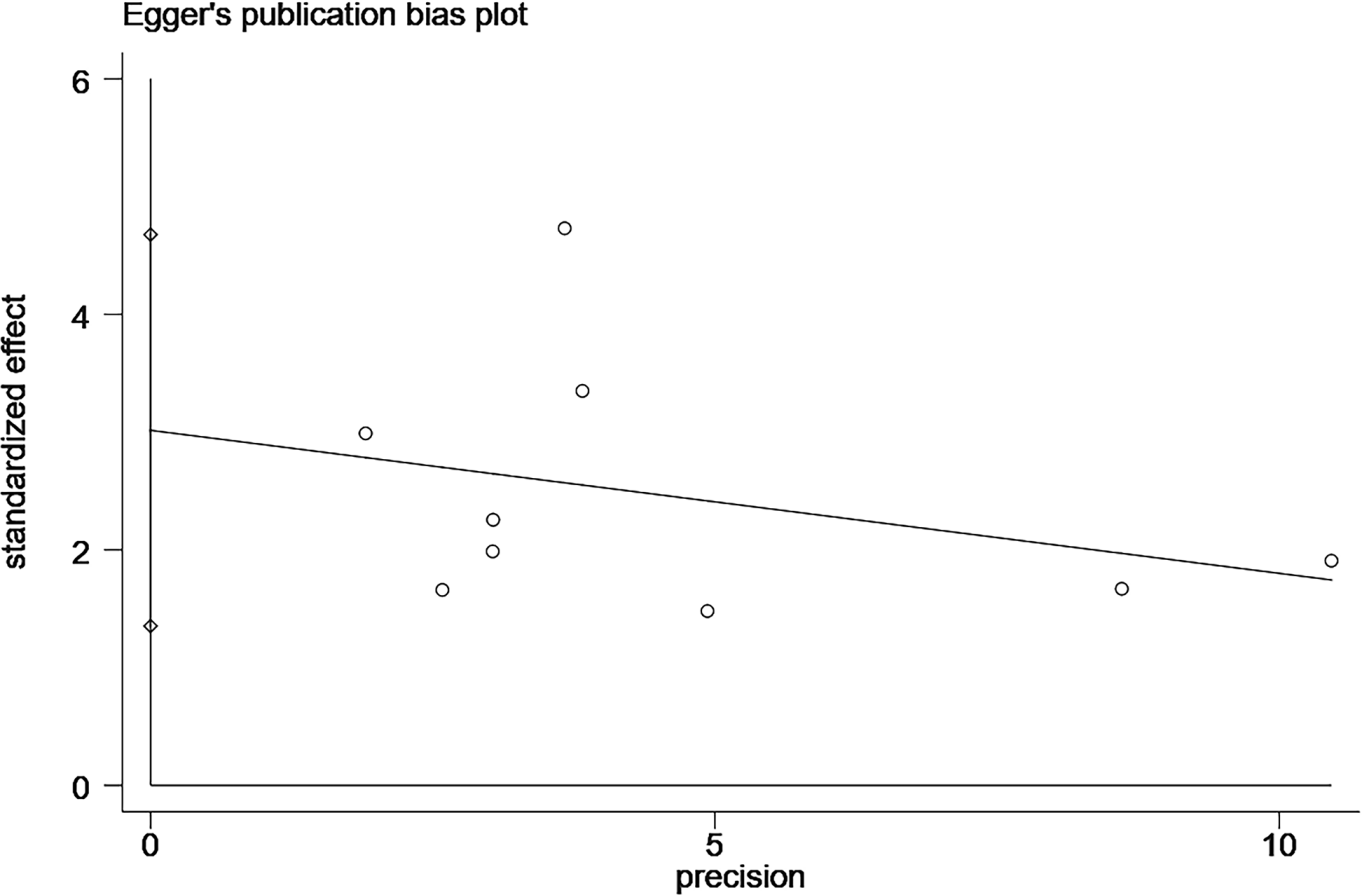
Plot of the Egger’s test for publication bias.

In the study related to the effect of elevated CgA on OS, we found publication bias using the Egger’s test (P=0.004; **Figure 4**; **Supplementary Figure S1**). A common source of publication bias is that our study sample was limited to published studies, but published studies often provide positive findings.

### Sensitivity analysis

**Figure 5:**
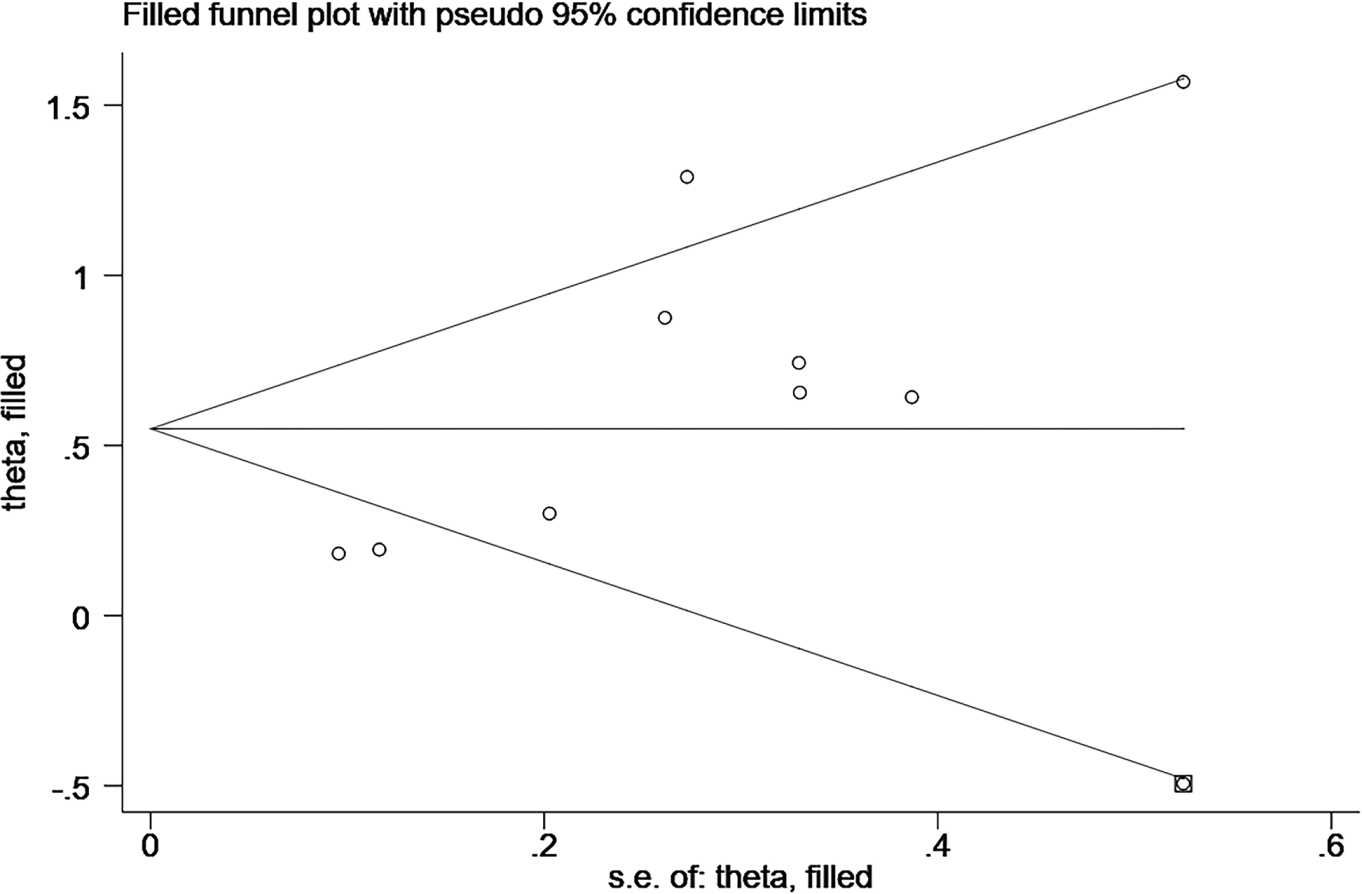
Sensitivity analysis by trim-and-fill method.

We performed sensitivity analysis using the trim-and-fill method, and the results after the trim-and-fill method were consistent with the results of our original study, all suggest a negative correlation between elevated CgA and OS (before trim-and-fill method HR=1.82, 95% CI: 1.38–2.41, p<0.001; after trim-and-fill method HR=1.73, 95% CI: 1.32–2.28, p<0.001; **Figure 5**; **Supplementary Figure S2**). It is suggested that our study did not affect the assessment of the results, although it was subject to publication bias.

## Discussion

Our study aimed to investigate the prognostic role of circulating CgA in prostate cancer, with a meta-analysis of 10 included studies (17, 19–27). Elevated circulating CgA is associated with poorer OS and PFS, but post-treatment CgA changes did not correlate with OS.

Four of the included literature suggest a negative correlation between elevated circulating CgA and OS (19, 20, 23, 24), three suggest a critical significance of elevated circulating CgA with shorter OS (21, 22, 26). And one article suggests no correlation between elevated CgA and shorter OS, but CgA showed prognostic significance in patients with a Gleason score ≥8 (HR=2.19, 95% CI: 1.16–3.85; p=0.0169) (17). Two study results suggested that elevated CgA was associated with poorer PFS (22, 24). One literature suggests that elevated CgA is critically associated with worse PFS (23). Combined with the results of our pooled analyses, we suggest that elevated circulating CgA is suggestive of poorer OS and PFS in prostate cancer, and the results of our meta-analysis and sensitivity analysis reflect rationality and reliability.

Of the total literature, two papers focused on the impact of post-treatment changes in circulating CgA on survival outcomes (25, 27). These two studies focused on the impact of post-treatment CgA elevation from baseline values on outcomes using patients’ CgA values at the time of the first specimen collection as the baseline value, and both studies suggested that post-treatment CgA elevation from baseline values did not correlate with OS. However, Hardenberg et al. (27) found that elevated CgA from baseline values after treatment was associated with worse PFS (HR=1.136, 95% CI: 0.999–1.291; p=0.052). Furthermore, patients with CgA elevations greater than 100% ULN had a trend toward lower OS and PFS in cycles 1 to 3, but it was not an independent predictor of OS in multivariate analyses. We believe that there are several factors that may account for the lack of correlation found between changes in circulating CgA after treatment and survival outcomes. The first is the heterogeneity of tumors, including differences in biologic characteristics and the degree of neuroendocrine differentiation. Prostate cancer is highly heterogeneous, and tumor cells from different patients have great differences in gene expression and biological behavior. Even after the same treatment, the regulatory mechanisms of CgA synthesis and release in tumor cells from different patients are different. Some tumor cells may proliferate and metastasis independently of CgA related biological processes. Therefore, the change of CgA level cannot reflect the progress of tumor, and thus cannot be correlated with survival outcome. In addition, the degree of neuroendocrine differentiation of prostate cancer varies, and only part of the tumor cells with neuroendocrine differentiation characteristics will secrete a large amount of CgA. If the proportion of neuroendocrine cells in the tumor is low, then the change of CgA level has limited effect on the overall tumor progression, and it is difficult to significantly correlate with the survival outcome. The second is the limitations of CgA detection, including the differences in detection methods and the lack of standardized detection methods. Different methods have differences in sensitivity and specificity, resulting in the accuracy and comparability of test results. Even if the samples from the same patient are tested in different laboratories, different CgA levels may be obtained, which interferes with the analysis of its correlation with survival outcomes. With regard to the impact of elevated circulating CgA on survival outcomes after treatment, the number of studies is small, and more studies are needed to further clarify this in the future.

Androgen deprivation therapy(ADT) is now widely used in prostate cancer treatment, and it was suggested that ADT induces neuroendocrine differentiation(NED) in prostate cancer (28). There are many different theories about why NED occurs. Han et al. (10) showed that expression of the transcription factor FOXA2 (which drives the transition of prostate cancer glands to the lineage plasticity) was significantly induced under ADT. Zhang et al. (29) suggest that ADT leads to the activation of CREB (cAMP response element-binding protein), which affects the neuroendocrine differentiation of prostate cancer through the CREB-EZH2-TSP1 pathway (EZH2: enhancer of zeste homolog 2, TSP1: thrombospondin-1, THBS1).

Liu et al (30) suggests that ADT induces leukemia inhibitory factor (LIF) expression, and LIF promotes neuroendocrine differentiation through activation of prostatic tumor promoter (ZBTB46). Enriquez et al (31) suggests that ADT leads to downregulation of the stromal cell protein SPARC in stromal cells, and that downregulation of SPARC causes stromal cells to release IL6, which is a NED inducer that drives neuroendocrine differentiation in prostate cancer. There are many more studies on the mechanisms by which NED occurs in prostate cancer, and it is not yet clear which mechanism predominates.

The presence of NED is often indicative of a poor prognosis, and the detection of circulating CgA reflects the neuroendocrine differentiation of tumor cells and has the advantages of simplicity and reproducibility, which can be considered as a biomarker of prognostic value and provides useful information for treating the disease. The biological role of CgA in prostate cancer progression includes the following aspects. First, CgA can promote the proliferation of prostate cancer cells by activating intracellular signaling pathways. Studies have found that CgA can bind to specific receptors on the surface of prostate cancer cells and activate the downstream PI3K-Akt signaling pathway. This signaling pathway plays a key role in cell growth, proliferation, and survival. When activated, Akt protein can phosphorylate a series of substrates and promote cell cycle progression, allowing cells to enter S phase from G1 phase, thereby accelerating cell proliferation (32). Second, CgA can enhance the invasion and metastasis of prostate cancer cells, which is reflected in the regulation of the expression of migration and invasion related molecules in prostate cancer cells. On the one hand, it can induce an increase in the expression of matrix metalloproteinases (MMPs). MMPs can degrade extracellular matrix components and create conditions for tumor cell migration and invasion (33). On the other hand, CgA may affect the expression of intercellular adhesion molecules. It can down-regulate the expression of E-cadherin, which is an important intercellular adhesion molecule. The reduction of E-cadherin expression will weaken the adhesion force between cells, and make tumor cells more likely to leave the primary tumor and migrate and metastasize (34). Third, it can regulate the tumor microenvironment. CgA has a regulatory effect on immune cells in the tumor microenvironment. It can inhibit the anti-tumor activity of immune cells, thereby helping tumor cells escape immune surveillance. For example, CgA can inhibit the proliferation and cytotoxicity of T lymphocytes and reduce their killing effect on tumor cells. At the same time, CgA can also promote the polarization of tumor-associated macrophages to M2 type. M2 type macrophages have immunosuppressive function and can secrete a variety of cytokines to promote tumor growth, angiogenesis and metastasis (35).

However, circulating CgA is affected by a number of diseases and medications, such as hypertension, heart failure, renal failure,and inflammatory bowel disease, and the use of proton pump inhibitors can cause an elevation of circulating CgA (36), which would be a confounding factor in the clinical work.

CgA is abnormally expressed in a variety of tumors, such as pheochromocytoma, small-cell lung cancer, medullary thyroid carcinoma, pancreatic islet cell tumors, and prostate cancers. Nowadays, many studies suggest that the increased release of CgA from neuroendocrine tumor cells is involved in the regulation of tumor growth and progression and that circulating CgA is a useful serum marker for diagnosing various types of neuroendocrine tumors (12, 37, 38). In Baudin et al’s (39) study, circulating CgA and NSE were measured in patients with neuroendocrine tumors (NET) and non-NET patients, and an analysis comparing them concluded that CgA appeared to be more reflective of tumor progression than NSE, suggesting that CgA should be used as a marker for screening patients with NET. In Campana et al’s (11) study, plasma CgA values were compared between NET and non-NET patients, and it was found that plasma CgA levels were higher in NET patients, and CgA levels were higher in patients with diffuse disease than in patients with localized disease.

There are also studies on the diagnostic value of circulating CgA in the diagnosis of prostate cancer, but there are differences between the results, with some studies showing that circulating CgA levels are higher in prostate cancer patients than in non-prostate cancer patients (40–42). However, other studies suggest that circulating CgA levels are not significantly different between the two and that detection of circulating CgA does not provide useful value in diagnosing prostate cancer (43–45). If the detection methods of CgA are standardized and the tumor heterogeneity is classified, CgA is likely to play a very important role as a key factor in the prognosis of prostate cancer. We think we can establish a prognosis evaluation system for prostate cancer combined with CgA, as follows: For low-risk prostate cancer, if the patient has normal CgA level, low PSA level (such as PSA < 10 ng/mL), Gleason score ≤6, and clinical stage T1-T2a, it can be judged as low-risk prostate cancer. Such patients have a good prognosis and a high 5-year survival rate. Active surveillance strategy should be considered, and CgA, PSA, prostate ultrasound and digital rectal examination should be regularly reviewed. If the CgA level was slightly elevated, and the PSA level was between 10-20 ng/mL, Gleason score was 7, and clinical stage was T2b-T2c, it was classified as intermediate-risk prostate cancer. The prognosis of these patients is moderate, and the treatment methods such as radical prostatectomy and radiotherapy can be selected according to the specific condition of the patient. The level of CgA should be closely monitored after treatment. If CgA continues to increase, the prognosis may be worse. If the level of CgA was significantly increased, accompanied by PSA > 20 ng/mL, Gleason score ≥8, clinical stage of T3-T4 or regional lymph node metastasis, it was considered as high-risk prostate cancer and highly suspected as neuroendocrine prostate cancer. These patients have a poor prognosis and usually require comprehensive treatment, such as surgery combined with radiotherapy, chemotherapy, and endocrine therapy. CgA was monitored dynamically during the treatment. If CgA did not decrease significantly or continued to increase, the treatment effect was not good, and the treatment strategy should be changed in time. More studies are needed to clarify the diagnostic role of circulating CgA in prostate cancer.

We evaluated the prognostic role of circulating CgA in prostate cancer through a systematic and comprehensive search of databases and performed subgroup and sensitivity analysis to demonstrate the reliability and stability of the results. Our study has some limitations. First, the sample size was not large enough, and second, CgA was measured differently between studies, which may lead to the presence of bias. Only two of the included studies focused on the impact of post-treatment changes in circulating CgA on outcomes. Therefore, more and larger studies may be needed in the future to confirm the prognostic role of circulating CgA in prostate cancer.

## Conclusion

Overall, elevated circulating CgA was associated with poorer OS and PFS in prostate cancer, according to our pooled results. This suggested that circulating CgA can provide prognostic value in prostate cancer. However, no correlation was found between post-treatment changes in circulating CgA and survival outcomes and more detailed and comprehensive studies with large sample sizes grouped according to clinical differences between patients are needed to further validate the prognostic role of circulating CgA in the future.

## Supplementary material

Supplementary Figure 1 Plot of the Egger’s test for publication bias.

Supplementary Figure 2 Sensitivity analysis by trim-and-fill method.
