# Supplementary figures and images for: Prognostic value of circulating Chromogranin A in prostate cancer: a systematic review and meta-analysis
Source: Front Oncol. 2025 Feb 5;15:1521558. doi: 10.3389/fonc.2025.1521558 (PMC11835686; doi:10.3389/fonc.2025.1521558)

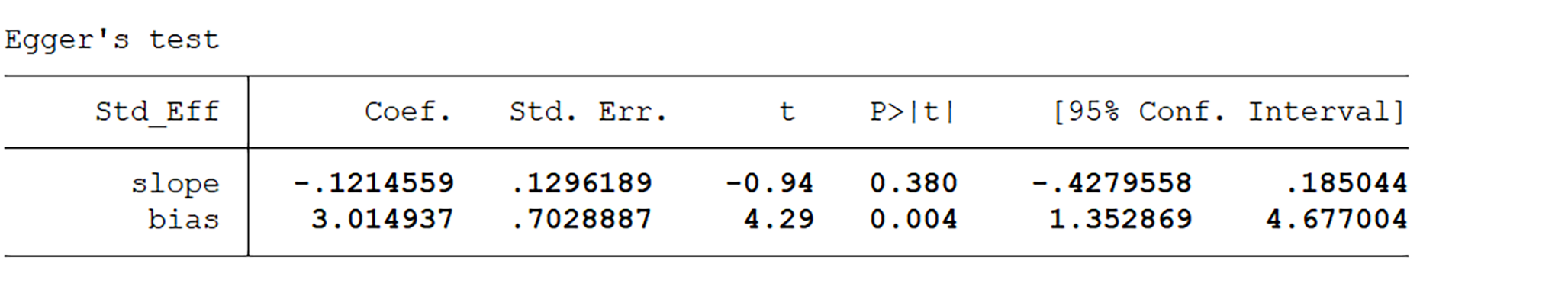

Supplement: Supplementary Figure 1 — Plot of the Egger’s test for publication bias. [file Image1.tif]

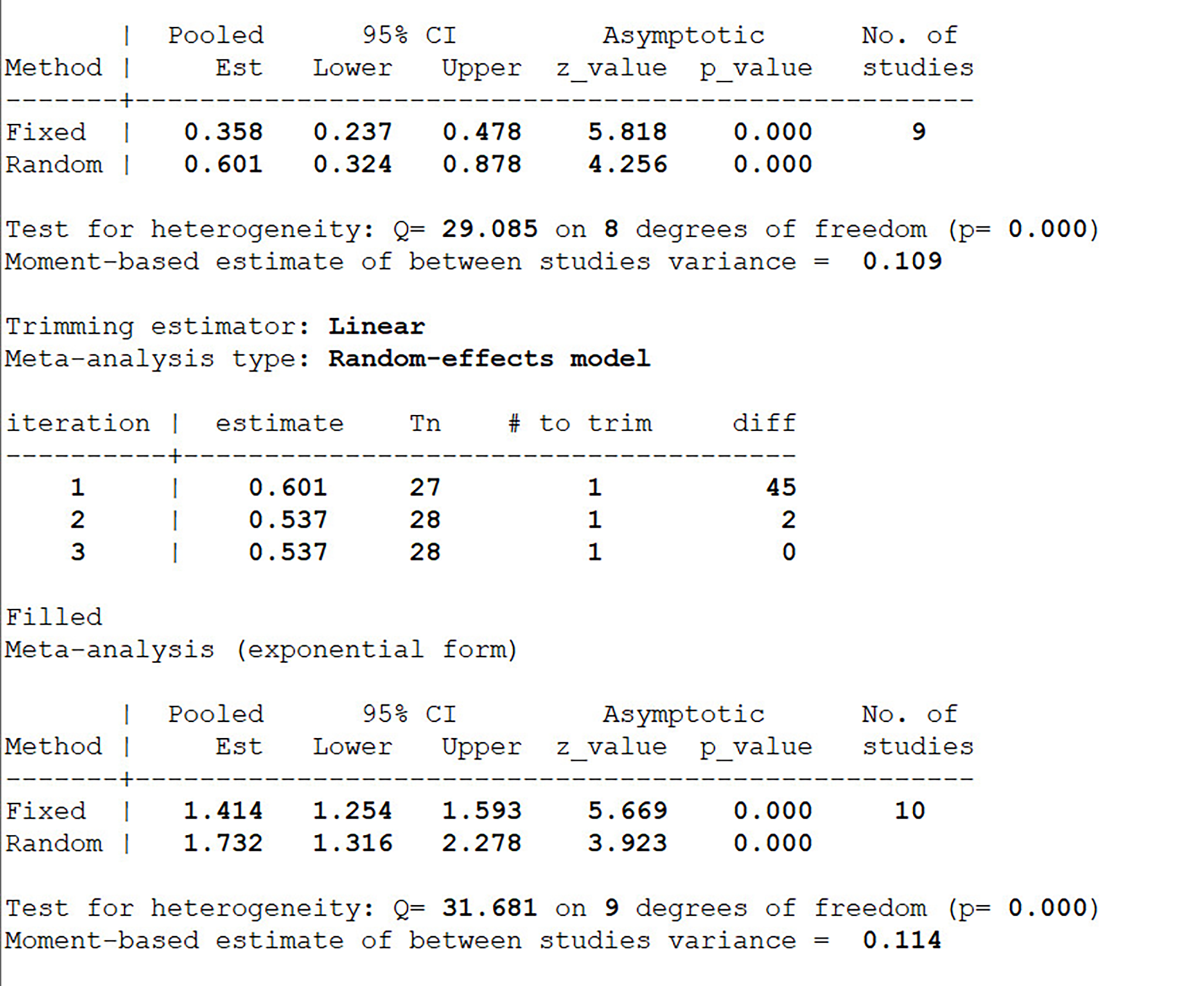

Supplement: Supplementary Figure 2 — Sensitivity analysis by trim-and-fill method. [file Image2.tif]
